# Prevalence of dyspnoea and usage of opioids in managing dyspnoea in advanced cancer patients: a longitudinal observational multi-centre study from India

**DOI:** 10.3332/ecancer.2022.1482

**Published:** 2022-12-02

**Authors:** Arunangshu Ghoshal, Anuja Damani, MaryAnn Muckaden, Pallavi Singh, Jayita Deodhar, Sumita Mohanty, Vidya Viswanath, Amit Grover, Priti Sanghavi, Sushma Bhatnagar

**Affiliations:** 1 Department of Palliative Medicine, Tata Memorial Hospital, Homi Bhaba National Institute, Mumbai 400012, India; 2 Department of Palliative Medicine and Supportive Care, Kasturba Medical College, Manipal Academy of Higher Education, Manipal 576104, India; 3 Regional Cancer Centre and SCB Medical College and Hospital, Cuttack, Odisha 753001, India; 4 Homi Bhabha Cancer Hospital and Research Centre, Visakhapatnam 530053, India; 5 Dr. D. K. Gosavi Memorial, Siddhivinayak Ganpati Cancer Hospital, Miraj 416410, India; 6 Department of Palliative Medicine, Gujarat Cancer and Research Institute, Ahmedabad, Gujarat 380016, India; 7 Department of Onco-Anesthesia and Palliative Medicine, Dr. BRA Institute Rotary Cancer Hospital, All India Institute of Medical Sciences, New Delhi 110029, India

**Keywords:** dyspnoea, prevalence, cancer, opioids

## Abstract

**Context:**

Breathlessness is one of the devastating symptoms experienced by patients with advanced cancer and can be very challenging to manage.

**Objectives:**

To find the point prevalence of dyspnoea in advanced cancer patients presenting to palliative care out-patient clinics, and the usage of opioids in palliation of dyspnoea.

**Methods:**

We conducted a prospective observational study among all consecutive patients presenting to the outpatient clinics of six cancer centres in India from different parts of the country. In addition to routinely documented demographic and clinical data from patient charts, study investigators collected information on the Edmonton Symptom Assessment System, Cancer Dyspnoea Scale (CDS) and European Organization for Research and Treatment of Cancer Quality-of-Life Questionnaire Core 15 Palliative Care. We calculated the prevalence of dyspnoea and documented the usage of opioids in palliation of dyspnoea using tests of differences across patient characteristics.

**Results:**

Between May 1, 2019, and April 30, 2020, 5,541 patients were screened for eligibility, and 288 were enrolled (48 patients from each of the six centres). We analysed the data of 288 patients, of which 36.4% had dyspnoea, with 28.5% with moderate to a severe degree (>4/10). Tiredness and loss of appetite were found to have associations with dyspnoea which were statistically significant on multivariate analysis. Standard palliative care management and routine usage of opioids preceded improvement in dyspnoea scores, CDS scores and quality of life scores throughout 7 days.

**Conclusion:**

Dyspnoea is a common symptom in advanced cancer patients, presenting to outpatient clinics, and routine documentation of dyspnoea with appropriate usage of opioids helps in mitigation.

**Key message:**

The article suggests that breathlessness is a common problem in advanced cancer patients and opioid prescription preceded symptom improvements in such patients.

## Background and rationale

Dyspnoea or breathlessness is an uncomfortable and subjective feeling of breathing difficulty, which may vary in character and intensity for individual patients. This is emphasised by the consensus definition of the American Thoracic Society and it also suggests that this experience may be influenced and individualised by various physiological, psychosocial, behavioural and environmental factors and their interactions [1].

Dyspnoea is one of the commonly reported symptoms in patients with metastatic cancers and the prevalence is higher in patients with advanced malignancies, especially towards the end of life [2, 3]. Progressive worsening of dyspnoea in a patient with advanced metastatic cancer is one of the markers of poor prognosis [4]. In a previous study in our centre, we found that the prevalence of dyspnoea in advanced cancers is as high as 44.37% [5]. The symptoms in cancer patients are often compounded with other symptoms and present in clusters. Some of the common symptoms that cluster with dyspnoea are fatigue, anxiety and depression, loss of overall wellbeing and functionality, leading to poor quality of life and higher caregiver burden [6, 7]. This also has major implications in terms of further patient-care, disease management, treatment, outcomes and survival [8, 9].

The clinical prevalence, quality and management of dyspnoea vary, and being a symptom, it is not measured often, leading to little data on this [10]. Dyspnoea can be highly subjective; thus, measurement requires asking the patient. In this study, participating palliative care units had existing processes to assess unidimensional assessments of dyspnoea for all outpatients as reported in a small sample from our clinic in a previous study [5]. In this study, we report the extent of the burden of dyspnoea in terms of its prevalence among a large sample of patients with advanced cancer in multiple regional centres across India.

## Methods

### Study population and methodology

We conducted a prospective observational multicentric study over 1 year between 2019 and 2020 in six high volume cancer centres from different regions of India. Tata Memorial Hospital (Mumbai) was the lead research site, and the others were Acharya Harihar Regional Cancer Centre (Cuttack), Homi Bhabha Cancer Hospital & Research Centre (Visakhapatnam), Shri Siddhivinayak Ganapati Cancer Hospital (Miraj), Dr. B.R.A Institute-Rotary Cancer Hospital – All India Institute of Medical Sciences (Delhi) and the Gujarat Cancer & Research Institute (Ahmedabad).

Data were collected in the palliative medicine outpatient clinics by the study investigators. The average time for completion of the protocol was 15 minutes. Study participants were adults with advanced cancer who have been informed of their diagnosis and are well enough to complete the questionnaire. Any participants who were suffering from severe mental or cognitive disorders, and with potentially curable diseases referred for early palliative care were excluded.

Consecutive patients with advanced cancer referred to the outpatient clinics of the palliative care departments, and those who satisfy the eligibility criteria were considered for the study. Positive screened subjects were explained about the study and study procedure in a language they best understand. Participants were given adequate time to consider participation and decide. The Participant Information Sheet was given to them, and all questions were clarified to their satisfaction. Consent was taken by the Principal or Co-investigators of the participating institutions. In case of non-participation, the reason for non-participation was recorded and routine care was offered. At the participating centres, routine palliative care is provided by specialised palliative medicine multidisciplinary team of physicians, nurses, rehabilitation therapists and social workers who coordinate care that focuses on the patient and family’s individual needs. The goal of such care is to provide the patient and their family with the best quality of life for as long as possible by assisting with the physical, emotional, social and spiritual stresses associated with life-limiting illness [11]. During the study, clinicians were able to start, adjust or stop other treatments for dyspnoea as per standard of practice according to clinical judgment. The study continued till the recruitment of a minimum sample size. The baseline data were recorded at presentation to the palliative care outpatient clinic. Those patients who were started on opioids as pharmacotherapy for breathlessness management were followed up on days 1, 3 and 7 to record the prescribing patterns and change in breathlessness scores.

### Study variables

On the day of study enrolment (day 0), data were collected from a chart review of routinely collected variables like age, sex, place of residence, family income (per month), educational status, contact number, marital status, primary caregiver, primary cancer diagnosis, cancer stage (at assessment), date of diagnosis of cancer, sites of metastasis, comorbidities (if any), treatment received for primary cancer, Eastern Cooperative Oncology Group score [12], symptom assessment on the Edmonton Symptom Assessment System (ESAS) [13, 14], current medications, and dosages. Two additional research questionnaires were used: Cancer Dyspnoea Scale (CDS) [15] and European Organization for Research and Treatment of Cancer Quality-of-Life Questionnaire Core 15 Palliative Care (EORTC QLQ-C15-PAL) [16] (details in Appendices). Follow-up, data were recorded on ESAS and CDS on day 1 and day 3 to record changes in symptom scores. On day 7, ESAS, CDS and EORTC QLQ-C15-PAL were recorded for change in symptoms and quality of life scores.

### Statistical considerations

Calculation of minimal sample size was based on literature citing the improvement in dyspnoea scores in advanced cancer patients with morphine and study site-specific adjustments for data variability. The predicted standard deviation of the Visual Analogue Scale (VAS) from the literature is 16 mm (the VAS ranges from 0 to 100 mm) [17, 18]. Thus, a minimum of 48 participants per centre could provide 80% power to detect a 10-mm difference in the scale, with α of 0.05, allowing for a 20% dropout rate [19].

We analysed data with descriptive statistics to find the point prevalence and regression methods with Network Visualisations [20] for the interrelationship of dyspnoea with other symptoms and quality of life. For modelling of the ESAS dyspnoea values over days 0, 1, 3 and 7, linear mixed-effects modelling was used to compare changes in scores between groups (with and without opioids) with adjustments for age, sex, study site and baseline dyspnoea level using CRAN lme4-package [21]. A first-order autoregressive structure was fit to adjust for autocorrelation among timepoints to account for individual variation in physiological or behavioural traits on repeated measures through time [22]. The model selection process used top-down strategy recommended by Zuur *et al* [23]. All analyses were performed on RStudio 2022.02.2 software [24]. All analyses used two-sided tests, and a two-sided* p* value of 0·05 or less was considered to be statistically significant.

The research was carried out in compliance with the Helsinki Declaration, and Ethics committee approvals were obtained from the Institutional Review Boards of all the participating centres and the trial was registered with the Clinical Trials Registry – India (CTRI/2021/10/037056).

## Results

**Figure 1.**
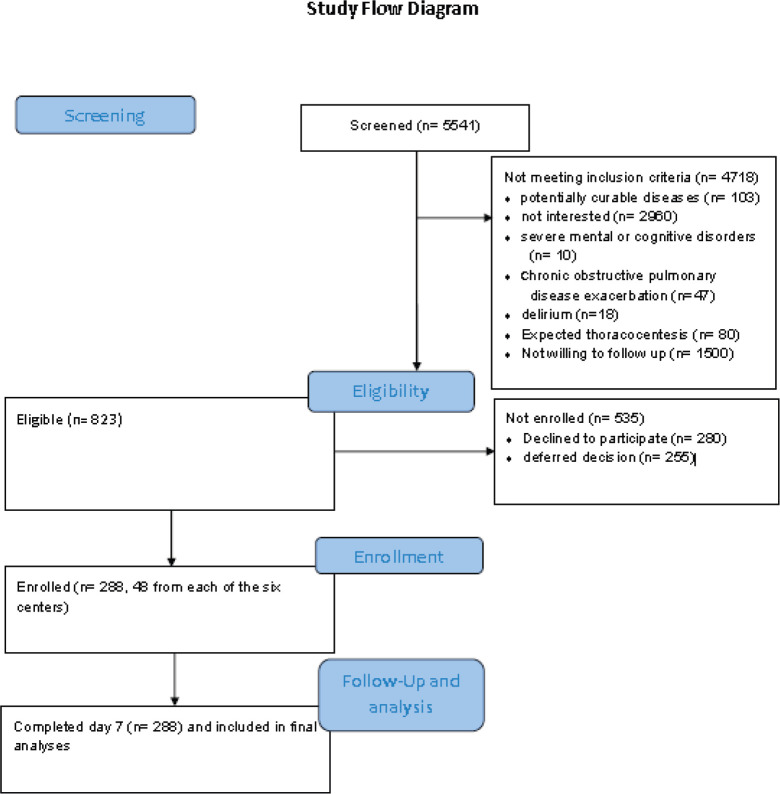
Study flow diagram.

Between May 1, 2019, and April 30, 2020, 5,541 patients were screened for eligibility. Two hundred and eighty-eight (35%) of 823 eligible patients were enrolled (48 patients from each of the six centres) (See Figure 1).

Screening for clinical dyspnoea as shown by the ESAS score identified the point prevalence of dyspnoea as 36.4%. Out of 288 patients who had dyspnoea, 206 (71.5%) had mild (ESAS score: 1–3), 44 (15.3%) had moderate (ESAS score: 4–6) and 38 (13.2%) had severe dyspnoea (ESAS score: >/= 7).

Study participants consisted of 56.2% men, with 54.2% in the age range of 41–60 years. The most common cancers were genito-urinary in 17.9%, breast in 15%, head and neck in 13.4% and gastrointestinal in 13.1% of patients. 24.3% of patients had comorbidities, with 22% having a chronic pulmonary disease.

On ESAS, the most common symptoms were loss of well-being in 75%, tiredness in 74% and pain in 71% of patients. The quality of dyspnoea was recorded by CDS with a median value of 6.2 on the effort score, 3.8 on the anxiety score and 6.6 on the discomfort score. On the EORTC-QLQ-C-15-PAL scores for functional scales were 46.6, for symptom scales was 54.6 and for global health status was 54.4 (Table 1).

**Figure 2.**
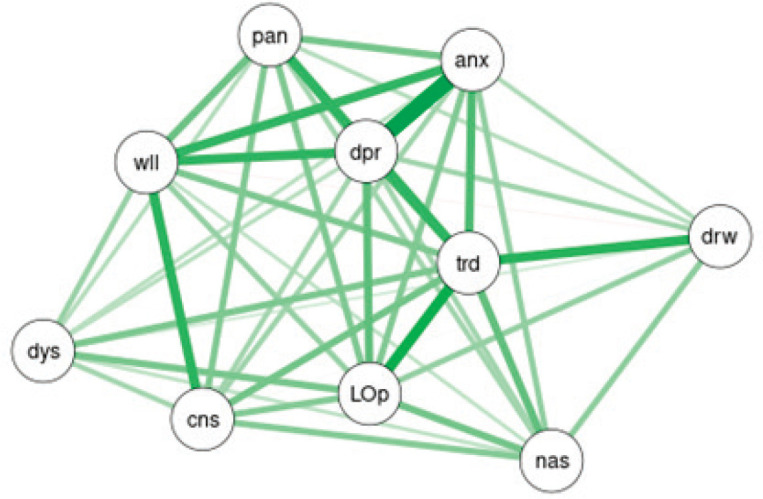
Network analysis of ESAS symptoms at baseline using force-directed plotting with Fruchterman–Reingold (normalised stress value = 0.18).

On multiple regression analysis between dyspnoea score and other items on ESAS, model validity is suggested by the *p*-value of the F-statistic is 1.312e-06, and significant predictor variables from the coefficients table are tiredness (t-statistic *p*-value = 0.04225) and loss of appetite (t-statistic *p*-value = 0.00475). The complexity of the interrelationships has been depicted in Figure 2 via network analysis of ESAS symptoms at baseline using force-directed plotting with Fruchterman–Reingold (normalised stress value = 0.18) (Table 2).

**Figure 3.**
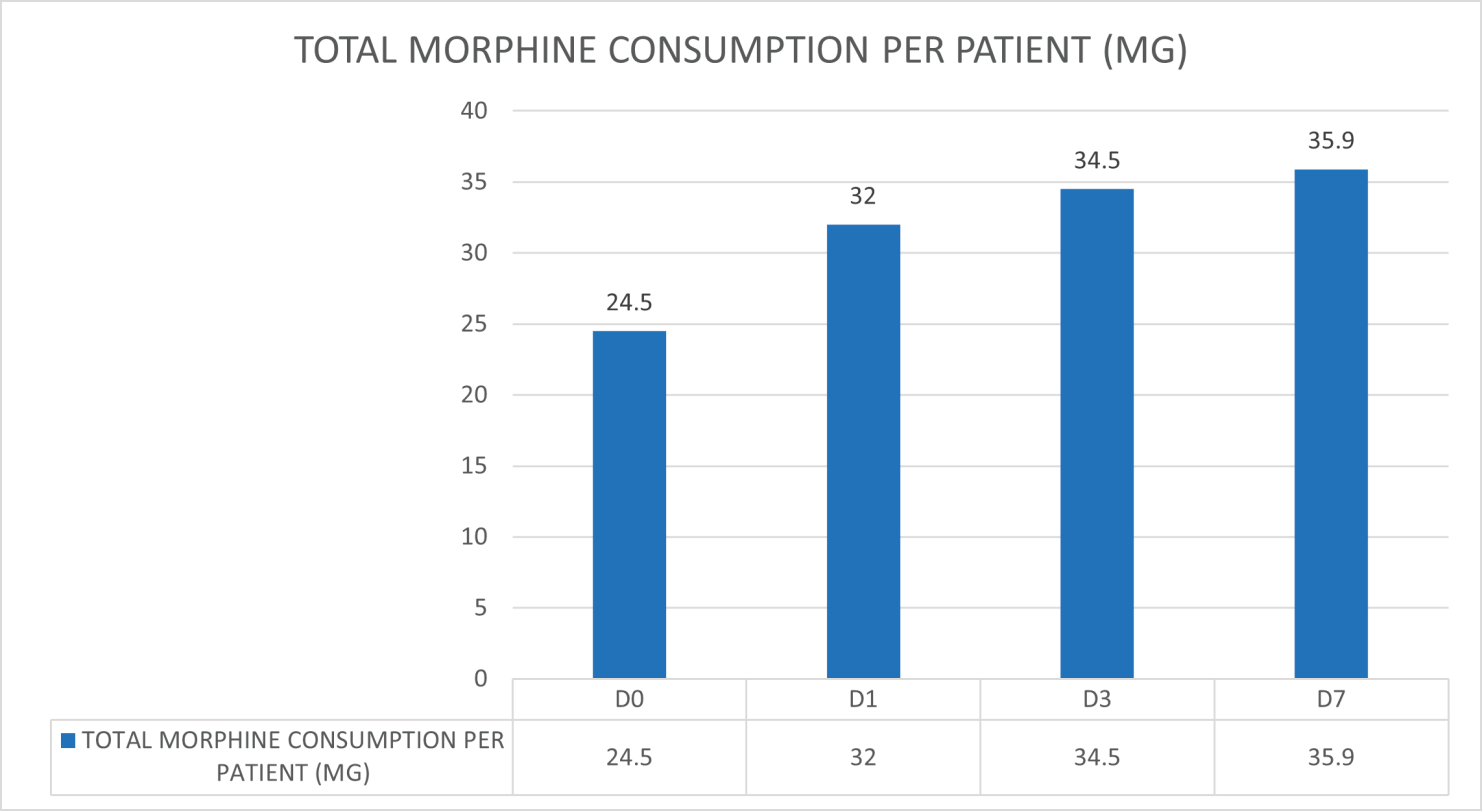
MEDD for patients on days 0, 1, 3 and 7, showing an increasing trend.

Of the 288 patients with dyspnoea, 112 patients received oral morphine. Of these, 90 patients received opioids for pain, and no additional opioid was given for dyspnoea. Twenty-two patients received opioids for dyspnoea. Opioid consumption per day was recorded as Morphine Equivalent Daily Dose (MEDD) for patients on days 0, 1, 3 and 7. There was an increasing trend for MEDD over 7 days (Figure 3). A Pearson product-moment correlation was run to determine the relationship between opioid usage in dyspnoea and clinical demographic parameters. There was a strong, positive correlation with total score in CDS, which was statistically significant (*r* = 0.706, *n* = 22, *p* = 0.005).

**Figure 4.**
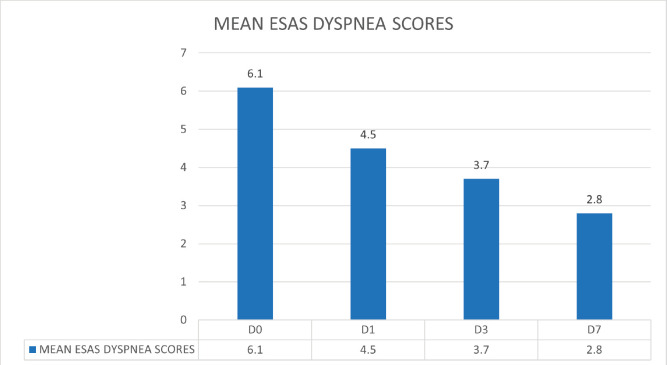
The mean dyspnoea scores on ESAS showing a downward trend from 6.1 on day 0 to 2.8 on day 7 post opioids and standard palliative care intervention.

The longitudinal results for change in opioid consumption, ESAS scores for dyspnoea, CDS scores and quality of life scores were calculated in 112 (38.9%) participants with moderate-severe dyspnoea, who received opioids and could complete all four observations on days 0, 1, 3 and 7. The mean dyspnoea scores on ESAS reduced from 6.1 on day 0 to 2.8 on day 7 post opioids and standard palliative care intervention (Figure 4).

**Figure 5.**
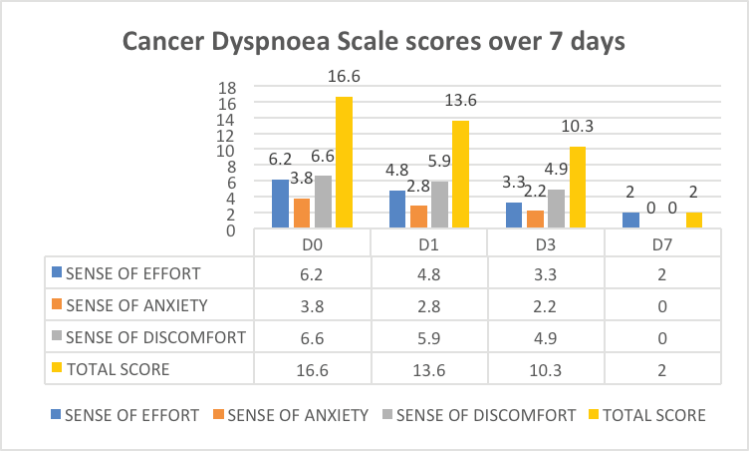
CDS scores in effort, anxiety, discomfort and total scores of breathlessness over 7 days.

There was a reduction in effort, anxiety, discomfort and total scores of breathlessness on CDS over a follow-up period of 7 days (Figure 5).

**Figure 6.**
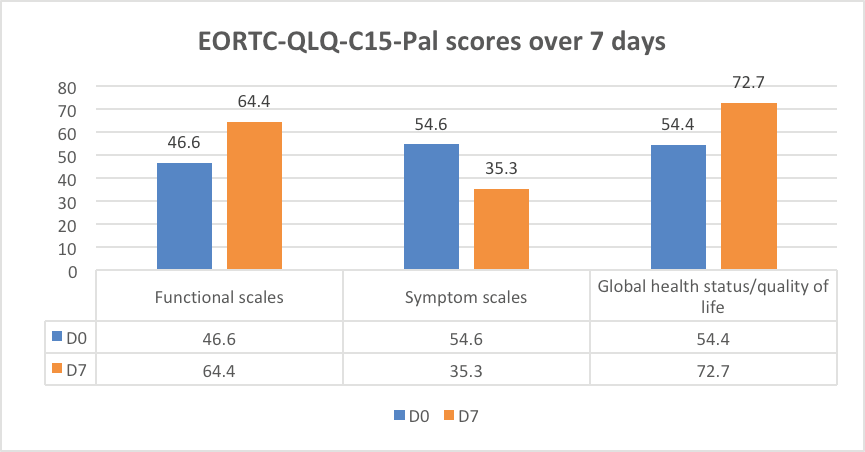
EORTC-QLQ-C15-PAL scores on day 0 and day 7, showing improvement in functional scores and global health status and a reduction in symptom scales.

Quality of life scores was recorded on day 0 and day 7 to identify the effect of dyspnoea management on functional scores (daily life activities), symptom scores and global health scores (overall wellbeing) of the patient. The improvement was noticed in functional scores and global health status and there was a reduction in symptom scales on day 7 on EORTC-QLQ-C15-PAL scores (Figure 6).

Overall, improvement in all the scores (ESAS dyspnoea scores, CDS scores, quality of life scores and MEDD) over 7 days showed a statistically significant result (*p* < 0.05). However, a mixed-model analysis showed that ESAS dyspnoea scores did not differ statistically between the groups (with and without opioids) by day 7 (Table 3).

A correlation between ESAS scores of dyspnoea and the feeling of well-being after the use of morphine (*n* = 22) (where 10 signifies Worst Possible Wellbeing) suggests a positive Pearson correlation coefficient of 0.83, *p* value of <0.001.

## Discussion

This is the first study determining the prevalence of breathlessness among palliative care outpatient clinic patients in major cancer centres in India from different parts of the country [25]. As many as 36.4% of patients attending the clinics present with breathlessness and one-quarter of them report severe breathlessness. It was heavily related to other symptoms, and thus was one of the most important symptoms in study participants.

Large-scale measurement of dyspnoea in clinics is feasible. Across all the participating centres, study investigators suggested that subjective assessment of dyspnoea and correct documentation was an important part of the patient-centred care approach. They also noticed that the process was less time-consuming, and that the unidimensional dyspnoea assessment measures using the 0–10 Likert scale were efficient for screening dyspnoea in busy outpatient settings. However, multidimensional assessments are preferred in specialised palliative care practice, which is essential for a better understanding of the factors contributing to the patient’s overall symptom experience [26]. The study results of this study were based upon the data obtained from the patients directly attending palliative medicine outpatient clinics over 1 year. In a study by Baker *et al* [27], the authors described similar findings and suggested that palliative care nurses can use unidimensional and multidimensional measures to assess and characterise breathlessness in busy clinical settings.

In this study, data collection and symptom assessment were done while the patients were seated for a while after arrival at the clinic. Hence, logically these ratings suggest dyspnoea in the non-exertional and resting condition of the patient. It was noticed that patients with cardio-respiratory system involvement due to cancer or other co-morbidities like chronic obstructive pulmonary disease, heart diseases and chronic kidney disease reported higher intensity of dyspnoea, with a score > 4/10. Also, those patients who reported pain score > 4/10 (*n* = 123), and fatigue score > 4/10 (*n* = 101) at the time of assessment had a 2.8- and 2.3-times higher prevalence of dyspnoea, respectively. These patients also reported higher intensity of dyspnoea. This depicts the impact of comorbidities and the presence of other symptoms on the patient’s overall experience of dyspnoea.

ESAS estimates the level of dyspnoea to examine the average symptom intensity over the past 24 hours. This recalled dyspnoea rating is subjective, as it varies depending on the amount of physical exertion and overall experience during the interval time, and physiologically represents cardiorespiratory demand about delivery [28]. Most patients reported that their experience of the worst dyspnoea was not related to the level of exertion. This should not be surprising, as the study sample comprised of cases with advanced cancers with a conglomeration of other symptoms related to dyspnoea.

The reported prevalence of dyspnoea in advanced cancer patients is 36.4% in this study, which is comparable to a meta-analysis that included more than 10,000 patients with advanced cancer, with 10%–70% of patients reporting dyspnoea [2]. This difference can be due to the difference in the assessment of dyspnoea. Most studies in the literature assessing the prevalence of dyspnoea have used the Medical Research Council (MRC) dyspnoea scale [29]. The MRC identifies the level of breathlessness based on the level of exertion that provokes breathlessness and is a more objective measure. We used more subjective assessment tools, to determine the overall perception of dyspnoea.

The American Society of Clinical Oncology in their 2021 guideline for management of dyspnoea in advanced cancer recommended the usage of systemic opioids for patients who derive inadequate relief from nonpharmacologic interventions [30]. In a dose-finding pilot double-blind randomised clinical trial by Hui *et al* [31] prophylactic fentanyl improved breathlessness and walk distance (pre–post analysis) compared with placebo. Our study design unfortunately does not allow us to claim that opioid usage led to alleviation of dyspnoea using a mixed-model analysis; however, we documented that the usage of opioids preceded dyspnoea alleviation here. This is significant for Indian settings, as apart from the fear of respiratory depression and accelerated death with the usage of opioids [32], there are barriers to opioid usage/availability like bureaucratic hurdles and sociocultural/infrastructure challenges[33].

Our study data are restricted to palliative care outpatient clinics, and thus do not include many patients who attend oncology clinics. In high-risk groups with cardiac and pulmonary comorbidities, literature reports an increased prevalence of dyspnoea, up to 50%–70% [34]. We noticed differences in the prevalence of dyspnoea based on patient demographics like gender and age. Both patient and clinician gender influence the expectation of dyspnoea and its treatment [35]. These findings were consistent with the findings of Santos *et al* [36], who reported a higher prevalence of breathlessness among female gender and elderly patients.

We used clinic data for research purposes with necessary approval, which is considered real-life data and superior to administrative data. All patients provided a numerical rating for ESAS symptoms including dyspnoea. Such ordinal scoring captures full information, rather than a binary yes/no. For binary response choices, a participant may need some specific intensity of breathlessness to respond ‘yes’ to the presence of sensation and this magnitude may vary from person to person in different situations [37]. Our analyses cannot account for biases related to reporting, perceived experience or documentation.

ESAS data were collected by research staff including trained nurses and doctors. The data represents the real-world scenario and can be related to practical experiences in outpatient clinics:

1) Dyspnoea, being a subjective experience, should be patient-reported. However, doctors and nurses use some objective measures for respiratory distress to supplement patients’ reporting of breathlessness. One of the reasons for this is perceived difficulty for patients to quantify the experience of breathlessness in a numeric scale or in cases where patient is unable to vocalise. Hence, it is worthwhile to use some objective measures of ‘observed respiratory distress’ in addition to the subjective reporting, to have more robust information.

For all the study participants who complete the reporting, we acknowledge that 10%–15% of participants may find it difficult to translate their perceived symptom experience to a numeric value. Ideally, such patients are ‘poor raters’ and should be excluded from the actual data analysis. We acknowledge the error in measurement that might have crept in due to their presence in the study population. The study investigators across all participating centres agreed with the fact that these ‘poor raters’ were the patients who are unable to numerically rate all the subjective experiences like pain, breathlessness and other symptoms. This is noteworthy while assessing the patients in routine clinical practice, to be aware of false reporting.

2) Ideally, dyspnoea assessment should be done at the first visit to the hospital, but such assessments are challenging and were out of scope of this study. The prevalence of dyspnoea as indicated in this study may not be the true representation of patients presenting to hospital with breathlessness. It might have been slightly under-estimated as some of the severely breathless patients, who are unstable at presentation, will be shifted to the emergency department upon arrival, to receive treatment before the study enrolment could be made. To help circumvent this coverage gap, both ESAS and CDS include an item for assessment of dyspnoea in past few days before presentation to hospital, recall for dyspnoea experience in this period is dependable.

## Conclusion

Dyspnoea is one of the most distressing symptoms in patients with cancer. In this study, a third of the patients presented with dyspnoea of moderate to severe intensity. Cancer-associated dyspnoea includes multiple causes ranging from cancer itself to multiple ongoing comorbidities, respiratory and cardiovascular causes being the major ones. Although dyspnoea is less prevalent than pain, it should be routinely assessed as it significantly adds to both physical and emotional suffering. It is evident that assessment and documentation of dyspnoea are easy, less time-consuming and can be accurately done by all healthcare professionals, including doctors and nurses and its management significantly impacts overall patient experiences. The usage of opioids preceded dyspnoea alleviation in this study. We recommend that dyspnoea assessment should be routinely included as a part of the patient care plan, and opioids should be opted for palliation of dyspnoea.

## Disclosures and conflicts of interest

The authors have no conflicts of interest to disclose. De-identified individual participant data underlying the results reported in this paper will be available, for 5 years after publication, to investigators for uses approved by the institutional review board of the Tata Memorial Hospital. Proposals should be directed to the corresponding author. Acceptance of a data access agreement will be required for access.

## Author contributions statement

AG, AD, MAM and JD contributed to the conception or design of the work; AG, AD, PS, SM, VV, AGr, PSa and SB helped in data collection; AG and AD contributed to data analysis, interpretation and drafting the article; AG, AD, MAM, PS, JD, SM, VV, AGr, PSa, SB helped in critical revision and drafting the final version of the article to be published.

## Figures and Tables

**Figure 7.**
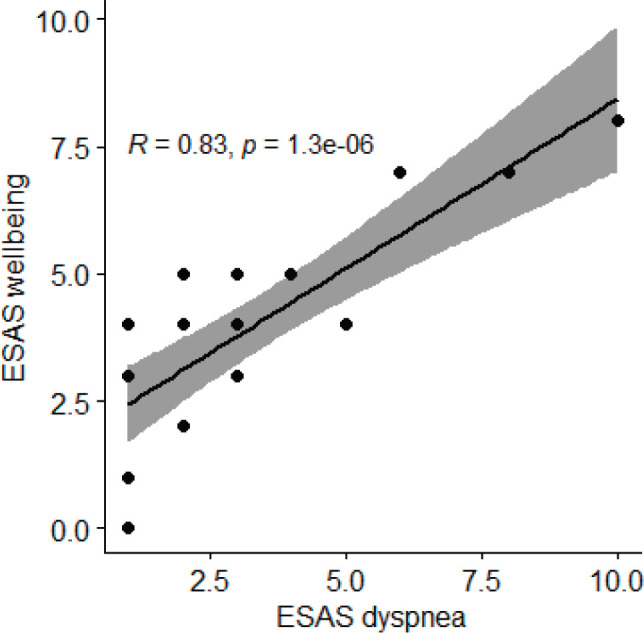
Correlation between ESAS scores of dyspnoea and the feeling of well-being after the use of morphine (*n* = 22).

**Table 1.** Demographic and clinical variables at baseline.

Variables	Description	Number (%) *n* = 288
Gender	Female Male	126 (43.75) 162 (56.25)
Age	18–40 years 41–60 years >60 years	56 (19.44) 156 (54.16) 76 (26.38)
Number of comorbidities	None 1 2 3 >4	218 (75.69) 49 (17.01) 17 (5.9) 3 (1.04) 1 (0.03)
Comorbidities (counts)	Chronic pulmonary disease	63 (22)
	Hypertension	130 (45)
	Depression	125 (43)
	Acute pulmonary disease	26 (9)
	Diabetes	9 (3)
	Chronic kidney disease	49 (17)
	Anaemia	112 (39)
Cancer type	Head and neck	39 (13.4)
	Thoracic	28 (9.6)
	Breast	43 (15)
	Hepatobiliary	18 (6.4)
	Gastrointestinal	38 (13.1)
	Genito urinary	51 (17.9)
	Sarcoma	9 (3.1)
	Skin cancers	8 (2.8)
	Central nervous system tumours	6 (1.9)
	Bone tumours	6 (2.1)
	Haematological	25 (8.6)
	Carcinoma of unknown primary	17 (6.1)
ESAS (0–10) median scores and point prevalence in %	Pain	5 (71)
	Tiredness	6 (74)
	Drowsiness	3 (16)
	Nausea	4 (31)
	Loss of appetite	4 (53)
	Shortness of breath	6 (36)
	Depression	6 (39)
	Anxiety	5 (36)
	Loss of wellbeing	5 (75)
	Others like constipation, sleep	4 (37)
EORTC-QLQ-C-15-PAL scores	Functional scales	46.6
	Symptom scales	54.6
	Global health status/quality of life	54.4
CDS median score	Sense of effort	6.2
	Sense of anxiety	3.8
	Sense of discomfort	6.6
24-hour morphine consumption in milligrams (MEDD)	Day 0	24.5
	Day 1	32
	Day 3	34.5
	Day 7	35.9

**Table 2.** Longitudinal comparison of scores day 7 versus day 0.

Test Statistics^a^									
Items	EORTC-QLQ-C-15-PAL			CDS				ESAS Dyspnoea score	MEDD
	Global health status	Symptoms	Function	Total score	Sense of discomfort	Sense of anxiety	Sense of effort		
*Z*	−4.953^b^	−5.454^c^	−4.728^b^	−6.738^c^	−6.642^c^	−6.227^c^	−6.336^c^	−6.041^c^	−4.234^b^
Asymp. Sig. (2-tailed)	<0.01	<0.01	<0.01	<0.01	<0.01	<0.01	<0.01	<0.01	<0.01

a Wilcoxon signed ranks test

b Based on positive ranks

c Based on negative ranks

**Table 3.** Mixed-model analysis of ESAS dyspnoea scores between the groups (with and without opioids).

	npar	Akaike information criterion (AIC)	Bayesian information criterion (BIC)	logLik	Deviance	Chisq	Df	Pr (>Chisq)
reduced.model (patients not using opioids)	8	3,987.1	4,003.7	−1,989.5	3,979.1			
Full.model (patients using opioids)	9	3,988.8	4,009.6	−1,989.4	3,978.8	0.2868	1	0.5923

**Figure:**
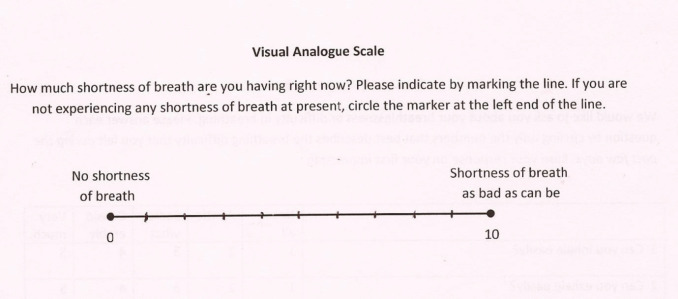
Dyspnea Visual Analogue Scale (VAS-D)- VAS-D (English)

**Figure:**
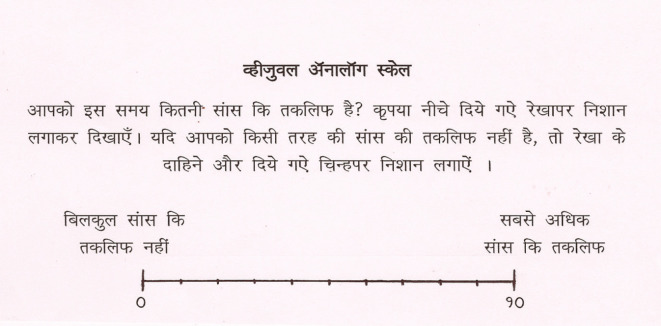
VAS-D (Hindi)

**Figure:**
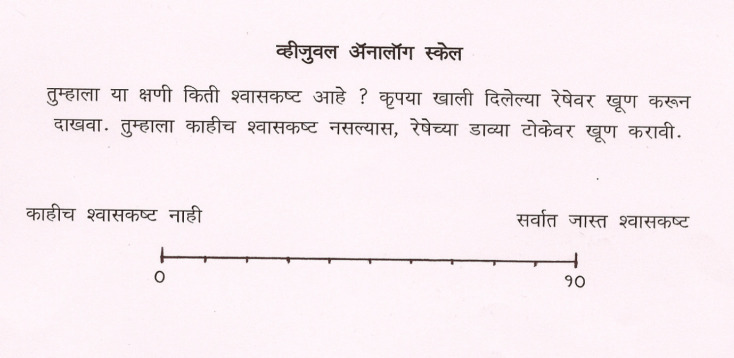
VAS-D (Marathi)

**Figure:**
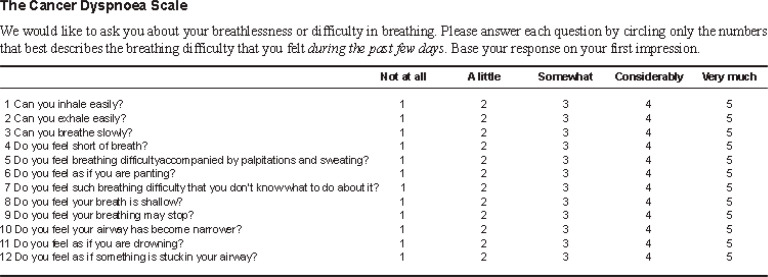
CDS-English

**Figure:**
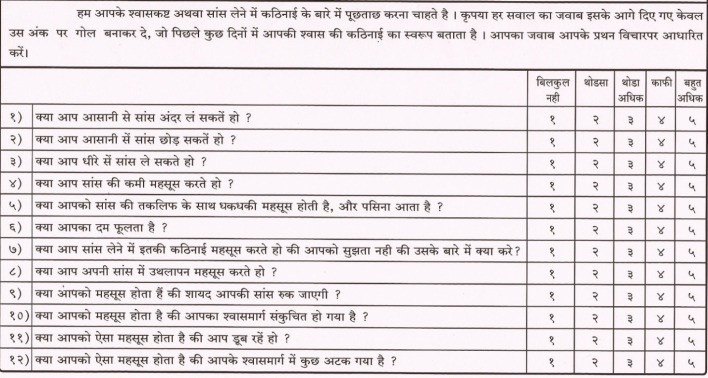
CDS-Hindi

**Figure:**
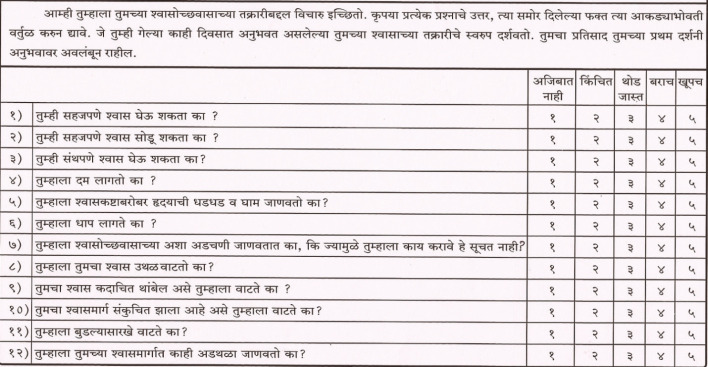
CDS-Marathi

## Appendices

### Appendix I Dyspnea Visual Analogue Scale (VAS-D)

It is a 10 cm uncalibrated horizontal line and has been translated and validated in Hindi and Marathi. The left end of the line is labeled “I Can Breathe as I Normally Do” and the right end of the line is labeled “I Can’t Breathe at all”. The patient’s mark was then converted to a score by measuring the distance from the left end of the line to the nearest 1 mm. Therefore, a dyspnea VAS score of 0 corresponds to the patient’s subjective feeling of “I Can Breathe Normally” and a dyspnea VAS score of 100 corresponds to “I Can’t Breathe at all.”

### Appendix II Cancer Dyspnea Scale (CDS)

The CDS is a 12-item self-rating scale validated to evaluate the multidimensional nature of dyspnea in cancer patients and has been translated and validated in Hindi and Marathi. The CDS consists of three factors: 1) “sense of effort” (5 items), 2) “sense of anxiety” (4 items), and 3) “sense of discomfort” (3 items). Each item is rated on a 5-point scale. The maximum score is 48 points. The higher the score, the more severe is the patient’s dyspnea.

### Appendix III European Organization for Research and Treatment of Cancer Quality of Life Core 15 Palliative (EORTC QLQ-C15 PAL)

EORTC C-15 PAL is a tool developed to measure the quality of life in cancer patients in a palliative care setting and has been translated and validated in Hindi and Marathi. It consists of 15 questions, which are transformed into two function scales (‘Physical Functioning’, ‘Emotional Functioning’), seven symptom scales (‘Fatigue’, ‘Nausea/Vomiting’, ‘Pain’, ‘Dyspnea’, ‘Insomnia’, ‘Appetite loss’, ‘Constipation’) and an ‘Overall quality of life’ scale. Patients should answer the questions according to their experiences during the previous week. Responses to 14 questions are given on a four-point Likert scale with 1 ‘Not at all’, 2 ‘A little’, 3 ‘Quite a bit’, and 4 ‘Very much’, the question to overall QoL allows answers between 1 ‘Very poor’ and 7 ‘Excellent’.
